# Book Reviews

**Published:** 1828-06

**Authors:** 


### CRITICAL ANALYSES.

Quæ laudanda forent, et quæ culpanda, vicissim

Illa, prius, cretâ; mox hæc, carbone, *notamus*.—Persius.

*Transactions of the Association of the Fellows and Licentiates of the King and Queen's College of Physicians in Ireland.* Vol. V. —8vo. pp. 576. J. Cumming, Dublin; Longman and Co. London, 1828.

As usual, in reviewing works of this nature, we shall confine ourselves in this department to those papers which contain general remarks, and place such of the cases as appear of sufficient interest in the Collectanea.

*On the use of the Nitrate of Silver in certain Affections of the Eye.* By Isaac Ryall, Esq.

Mr. Ryall remarks that there is no article in the materia medica so frequently useful in affections of the eye as the nitrate of silver; a circumstance, however, which has led to its very general adoption and too indiscriminate use. The object of the present paper is to draw a line of demarkation between those cases in which it is advantageous and those in which it is hurtful, and the author speaks, in succession, of those conditions in which its application is proper.

The first of these which he mentions is *ulcer of the cornea*, a termination of ophthalmia extremely common among those who apply for relief at the Eye Institution in Dublin. Frequently after protracted, and sometimes after mild, inflammations of the cornea, an "infinite number of small superficial ulcers" make their appearance upon it. For this form of the disease, Mr. Ryall recommends a solution in the proportion of two grains of the caustic to one ounce of distilled water; but if the ulcers do not readily heal, the strength of the solution is to be increased; and, in either case, the remedy is best applied by injecting into the eye. When the ulcers are deeper, the caustic is still more imperatively required, and, besides the injection, the ulcer itself must be touched either with a camel's-hair pencil dipped into a concentrated solution, or else with a piece of the solid caustic brought to a fine point; dropping a little oil of sweet almonds into the eye after each application. This is to be repeated on each separation of the eschar, till the ulcer assumes a healthy aspect.

Mr. Ryall, generally speaking, disapproves of mercury, recommending light nutritious diet and mild tonics. The vinous tincture of opium, so much used, he thinks a very injudicious application.

*Pustule* most frequently appears at the junction of the sclerotica and cornea. If the accompanying inflammation be severe, the usual antiphlogistic means are to be adopted; while the ulcerative process will be restrained, if not removed, by a few touches of the strong solution of caustic. When the disease runs into *sloughing of the cornea,*

"As an external remedy in this dangerous state of the eye, there is none so grateful, or withal so efficacious for throwing off the morbid parts, as a solution of the nitrate of silver in the proportion of eight grains to the ounce of distilled water, briskly injected against the slough three or four times a day. But, to obviate and arrest the progress of this serious termination of disease, we must rest our chief reliance on the extract of cinchona, so justly praised by the late Mr. Saunders. Opportunities are afforded to me but too frequently of giving this medicine a fair and full trial, in the cases of young infants born for the most part in the Lying-in Hospital; and I assert that, greatly as I had been prepossessed in its favor, it far exceeded my expectations, since both shape and function were preserved to an extent I dared not to hope for, in several instances where death of the exterior laminæ of the cornea, or of a segment of its entire thickness, had already taken place, and when the eye had presented the appearance of a disorganised mass. I have also lately used the sulphate of quinine in sloughing cornea with manifest advantage." (P. 7.)

When either ulceration or sloughing have made a cavity, through which the *protrusion of the iris* takes place, the nitrate of silver "is our chief, perhaps our only, resource." The early use of the belladonna, to prevent adhesions between the iris and adjacent parts, is particularly enjoined.

*Nebulous cornea,* which results from protracted inflammation, often proves extremely troublesome, and the opacity being sometimes so great as scarcely to permit the pupil to be seen, and occasionally the cornea even assumes the toughness and shining whiteness of cartilage. The following is the treatment recommended by our author:

"The application of leeches to the inner surface of the lower palpebra, the exhibition of purgatives, and precaution against strong liquors and cold, are the first objects to be attended to. If granulations exist on the palpebræ, they are to be removed by the means hereafter to be mentioned under the head of "granular palpebræ." After the vessels shall have been well emptied by the daily application of two leeches to the conjunctiva of the lower eyelid, and a few purgatives have been administered, a circle, or as large a segment of one as will include the opaque portion of the cornea, is to be described with the pencil-pointed caustic on the sclerotic, at about two lines distance from its junction with the cornea. After the eschar has sloughed off, ulceration is to be kept up for some time by the same means, care being taken to subdue any excessive inflammation which may be thus produced. The solution of the nitrate of silver, varying according to circumstances in the proportion of from two to six grains to the ounce, is to be frequently injected into the eye, and the ointment of the red oxyde of mercury applied every night to the tarsi, a portion of which may be allowed to be diffused over the surface of the eye-ball. In some of the worst cases of nebulous cornea, the Liquor Subacetatis Plumbi has been dropped into the eye twice a-day with great advantage. Absorption of the effused lymph, and contraction of the vessels, were speedily produced. It must be acknowledged, however, that these are not, in the majority of cases, its gratifying results. It is the practice with some to cut off, with a pair of curved scissors, a portion of the conjunctiva concentric with, and about two lines distant from, the cornea, and to continue the excision as far around the circumference of that tunic as its extent of opacity may require. The turgid vessels are by this plan emptied; but it has been found, when adopted in an irritable state of the organs, to create excessive inflammation and a new production of vessels. For the relief of that truly distressing attendant of this and other forms of protracted ophthalmy, profuse secretion from the lachrymal and tarsal glands, the dilute sulphuric acid, in the dose of from twenty to thirty drops in a glass of water twice or thrice a day, will prove of singular benefit; and it is probable that, if combined with the sulphate of quinine, its virtue would be still further enhanced." (P. 10.)

Mr. Ryall thinks that the use of the nitrate of silver has been much abused in the early stage of *albugo*. The re-absorption of the lymph producing it he thinks is best promoted by topical bleeding, and alterative doses of calomel, antimony, and opium. When, however, these fail, after a fair trial, then recourse must be had to a solution of "from four to eight grains of the nitrate of silver to one ounce of distilled water," to be used twice or three times a day; and a little of the weak ointment of red oxyde of mercury every night.

A few remarks are made on some other affections in which the caustic is of use, particularly Granular Palpebræ and Ectropion; but there is nothing particular in the mode recommended.

*Observations on the use of Ipecacuan in Menorrhagia.*
By J. OSBORNE, M.D.

Dr. OSBORNE thus states the results of his experience:

"I began the use of ipecacuan by ordering a scruple to be taken as an emetic at night, and I generally directed an acidulous saline purgative to be administered the following morning. The effect produced exceeded my most sanguine expectations. The discharge either ceased within twenty-four hours, or was so much diminished that no more remedies were necessary to insure its entire removal. In some few cases it recurred within a short time, but, when this did happen, it was only necessary to repeat the emetic once or twice in order to produce a permanent effect. I met with a few individuals in whom the discharge continued with little alteration after the first emetic, but with these I had only to repeat the remedy on the following night; and in one case alone three emetics were taken before the desired effect was produced." (P. 20.)

### *Observations on the Peripneumonia of Children.*
By Thomas Cuming, M.D.

Dr. Cuming is assistant physician to the "Institution for the Diseases of Children," a situation which has afforded him extensive opportunities of witnessing the disease of which he treats.

He states that peripneumonia is common in children of all ages, from a few days up to nine years, but that those from nine months to two years old are most obnoxious to it; a circumstance which he attributes to dentition, as predisposing to other disease.

"In some instances it commences suddenly, and without any assignable cause. It has sometimes happened that the child has gone to bed in perfect health, and risen next morning with all the symptoms of the disease distinctly marked. Such cases, however, are rare. In general a trifling cough, with other slight symptoms of catarrh, precedes, by a day or two, the complete formation of the disease. When fully formed, the symptoms by which the disease is characterised are a hurried, laborious, and wheezing respiration; a frequent, short, and dry cough, and a greater or less degree of fever. With the fever are combined an extreme degree of restlessness and impatience, moaning, starting out of sleep, and aversion to be moved. The countenance, though occasionally flushed, is for the most part extremely pallid, sometimes sallow; and, as the disease advances, it frequently assumes a mottled livid hue, and becomes in some cases swollen and œdematous. When the progress of the malady cannot be stopped, the breathing becomes more hurried and laboured, and the wheezing amounts in many cases to rattling. A state of drowsiness and prostration succeeds to the state of restlessness and pervigilium, which had existed in the beginning; the cough is suspended; the pulse becomes imperceptible; the extremities cold; and, in the course of eight or ten days from the commencement of the attack, death generally takes place by suffocation.

"With regard to the respiration, the average natural frequency of which, in an infant under twelve months, is about thirty in the minute, I have frequently observed it to be sixty, seventy-eight, eighty, ninety, and 102 in the minute; and in one case, that of a child six months old, I counted no fewer than 118 inspirations in the minute.

"When the respiration comes down from the frequency above mentioned to fifty-four or forty-eight in the minute, it generally indicates that the disease will terminate in recovery.

"In some instances there is an intermission of the breathing,—that is, for three or four inspirations and expirations it will be of natural frequency, then for about the same number it will be double or triple this frequency, and then again it will become as slow as at first.

"Although in almost every instance, as the disease advances towards its fatal termination, the breathing becomes more and more hurried, I have observed in one or two cases, in which in the beginning the respiration had been excessively hurried, that it fell to near the natural frequency a short time before death.

"In addition to its frequency, I have mentioned that the breathing is laborious. This state of the respiration is abundantly evident from the heaving of the chest, and the alternate dilatation and contraction of the alæ nasi; symptoms which are never absent when the breathing is much oppressed. In some instances, towards the close of the disease, the head is violently retracted, while the lower jaw is depressed at each inspiration, and the patient lies for a considerable time with his mouth open and gasping for breath before death puts a period to his sufferings.

"The respiration, though generally wheezing, is not always so. In some cases there is no wheezing at any period of the disease; and in others, though the breathing is loud and audible, the sound seems to proceed rather from some impediment offered to the air in its passage through the nostrils, than from mucous accumulation in the bronchia. It is difficult, by description, to convey an accurate idea of the sound to which I allude. I would call it a *dry* sound, in contradistinction to the other, which may be termed *humid*. It seems to be owing to a straitening of the air-passages, occasioned by the inflammatory turgescence of the membrane by which they are lined. When secretion takes place, this turgescence is in a great degree removed, and then the sound, from being dry, becomes moist and wheezing.

"In some instances the sound emitted in respiration seems to be a compound of the two sounds already mentioned, and to proceed partly from nasal and partly from bronchial obstruction. In the great majority of instances, however, the sound is distinctly wheezing, varying in degree from that of simple crepitus up to the loud and mucous rattle.*

* "The sound here described, though in most cases abundantly evident to the naked ear, is heard with much more distinctness when we have recourse to the employment of the stethoscope. By means of this instrument we are

"The cough, which is generally short and dry, is for the most part frequent at the commencement, becomes less so as the disease advances, and towards the close it is occasionally suspended altogether. A return of the cough, after it has been for some time suspended, is always to be considered as a favorable symptom." (P. 30.)

The stomach, at the commencement, is occasionally irritable, but becomes torpid as the complaint advances, so that the strongest emetics will then fail to act. This insensibility is not confined to the stomach, but is extended to the skin, which it is frequently impossible to vesicate by a blister. When the attack terminates fatally, the event generally occurs about the eighth or tenth day. When recovery takes place, the disease seldom lasts above a week. On post-mortem examination,

"The morbid appearance most frequently met with is an increase in the solidity of the lung, varying in degree from that of the slightest sanguineous congestion up to complete hepatization. This increase of solidity or induration is not equally great in every part of the lung. The inferior and posterior portion of the lung is in general the part principally affected; and it frequently happens that, while the upper portion is in a healthy state, or merely a little more congested than natural, the inferior portion is completely hepatised. It would appear as if the morbid process, commencing in the lower part of the lung, had completed its course there before the superior portion had advanced beyond the stage of sanguineous congestion. By hepatization, I mean that state of the lung which is characterised by a purplish red colour externally, a bright red colour and granular appearance when the lung is incised, and a total absence of crepitus, the lung feeling firm and solid, and sinking in water. This state corresponds with the second degree of inflammation of the lung, as described by Laennec. The first degree of inflammation, according to the same author, is the state of sanguineous congestion above mentioned. Laennec's third degree of inflammation, or that which is characterised by a purulent infiltration of the lung, I have not had an opportunity of observing. When the first degree of inflammation, or that of sanguineous congestion, prevails, it is generally combined with more or less of serous effusion into the interlobular tissue of the lung; but, where the lung is hepatised, also enabled to ascertain whether the inflammation is confined to the mucous membrane; for, when the substance of the lung is affected, this is sufficiently indicated by the indistinctness or absence of the respiratory murmur in that portion which is the seat of disease. If, in the exposition which I have given of the symptoms of this disease, I have not mentioned the stethoscope, my omitting to do so has not arisen from any want of confidence in the indications afforded by this instrument, the value of which I can duly appreciate; but solely because I have not yet attained to that precision in its use which would justify me in speaking of it as a means of diagnosis."

its section appears dry and granular, and very little serous or any other kind of effusion escapes.

"Although an increase in the solidity of the lung is the morbid appearance most frequently, indeed constantly observed, yet along with this, in almost every case, is combined more or less of inflammation of the mucous membrane of the bronchia. In some cases the inflammation extends to the trachea, the mucous membrane of which is more vascular than natural, and smeared with a tenacious mucus. In a few instances which fell under my observation, the trachea and bronchia were both highly inflamed, (two were cases of genuine croup,) and contained a considerable quantity of purulent mucus. Where the bronchia and air-cells are filled with mucus, the lungs collapse imperfectly, and nearly fill the corresponding cavities of the chest. In two cases, in which no trace of inflammation could be detected in the trachea or bronchia, the air-cells were filled with a purulent mucus, which exuded in abundance on making a section of the lung. In only one case, and that was a case of pertussis combined with inflammation of the substance of the lungs, which terminated fatally by convulsions, was the mucous membrane of the trachea, bronchia, and air-cells perfectly free from apparent disease.

"The more intense the inflammation of the mucous membrane, and the more considerable the effusion into the bronchia, the less in general is the induration of the lung, and vice versâ. In the cases of tubercular disease above mentioned, the tubercles co-existed with induration of the substance, and inflammation of the mucous membrane of the lungs." (P. 40.)

In this, as in other diseases, the danger is in proportion to the intensity of the symptoms; but those which may be pointed out as denoting urgent danger, are turgidity of the countenance, with coma and a rapid intermitting pulse;—indeed, under these circumstances, the event is almost invariably fatal.

In the treatment, three indications are laid down, all of which have reference to the effusion which takes place into the respiratory organs. 1. To arrest the inflammation, and thus prevent effusion; 2, to moderate this, if its entire prevention be not possible; 3, to promote the absorption and expectoration of whatever has been effused.

If we are fortunately called in at the very commencement of the disease, the first of these indications may sometimes be fulfilled; and blood-letting is the great remedy to be employed. Dr. Cuming says, it is a mistake to suppose that children do not bear bleeding well, or that their ailments do not require it. The earlier the bleeding is employed, of course the better chance there is of its not being required again, and the following is our author's estimate of the proper quantities:

" In a child about two years of age, from three to four ounces may be abstracted; and when the age is above four, about five, six, or eight ounces may be drawn, according to circumstances. In an infant under six months, though general blood-letting may often be required, the application of three or four leeches to the back of the hand or foot will for the most part answer the purpose where a vein, which is frequently the case, cannot be found. It is better to apply the leeches to the hand or foot than to the thorax, for, when they are applied in the latter situation, it is difficult to stop the bleeding after they have fallen off; and instances have occurred in which a fatal hemorrhage has been the consequence of a continual oozing from four or five leech-bites. When they are applied to the hands or feet, the bleeding can be easily stopped by placing compresses of lint over the bites, and securing them, as after the operation of V.S., by a bandage. As far as my observation goes, leeches applied to the extremities are nearly as efficacious in removing local inflammation in infants, as when applied in the vicinity of the part affected. They seem to produce the effect of a general blood-letting; as the face and lips become pale, the pulse faulters, and syncope occasionally takes place, followed by vomiting. These effects are apt to be produced when general V.S. is carried to a considerable extent, and sometimes a state of nervous agitation and general commotion is induced, which, if not speedily removed, may terminate in death. The best remedies in a case of this kind are the horizontal position, cool air, and a drop or two of the tincture of opium." (P. 49.)

Dr. Cuming remarks, that he has seldom seen the blood cupped or buffy in this complaint. As auxiliaries to blood-letting, emetics and purgatives are recommended. Of the emetics, our author gives the preference to the antimonials, and he thinks that the apprehensions of Dr. Clarke on this subject are groundless. Of the various purgatives, calomel and jalap are those which he most approves. " To a child between six and twenty months, we may give a grain of calomel, with four or five of jalap, and one of ginger, for a dose." After the preceding means have been premised, a blister is recommended to be applied between the shoulders or to the chest, and our author seems to place considerable confidence on this class of remedies; for he states that, after both the chest and back have been blistered, he has seen benefit from the "application of a blister to either side." When the debility is pressing, and suffocation is threatened, stimulants are recommended: of these he prefers the carbonate of ammonia, and in this our own experience fully corresponds with his.

*Observations on Cow-Pox, and on the Necessity of adopting Legislative Measures for enforcing Vaccination, in a Letter to* Mr. THOMAS BROWN, *Surgeon, Musselburgh, containing Remarks on his " Letter to the Right Hon. the Earl of Liverpool, concerning the present State of Vaccination."* By HENRY EDMONSTON, A.M. Surgeon, Newcastle upon Tyne.—8vo. pp. 156. Longman and Co. London, 1828.

THE author of this little volume very candidly informs us in his preface, that we need not look " for any thing in the shape of original discovery" upon the subject upon which he treats. " Dr. JENNER begun, and Dr. BARON has ended, by leaving nobody any thing to do." If nothing new can be expected upon the subject of vaccination, there are still many points concerning it which are open to discussion, which require to be definitively determined, and the consideration of which cannot be deemed either tedious or uninteresting.

We shall pass over as much as possible the critical tartness with which Mr. EDMONSTON comments upon the doctrines of the gentleman he addresses, and confine ourselves to his observations upon the material points of the subject. We beg, however, not to be understood as making this observation in the tone of reproof for any undue severity on the part of Mr. E. He evidently feels warmly, and writes with corresponding energy. We think also he complains with justice not only of the matter contained in the letter which calls forth his strictures, but of the mode in which that letter was addressed to Lord Liverpool. Mr. BROWN, it appears, has long been the supporter of the proposition " that the vaccine influence over the human body, as enabling it to resist small-pox contagion, is feeble, partial, and temporary." These are, indeed, formidable objections to the practice, and such as the general voice (to which we may add our own experience, which has not been trifling,) decidedly opposes.

Although Mr. Edmonston's Letter is addressed to a particular individual, its application is meant to be general to those who adopt the opinions of Mr. Brown.

Upon every point connected with vaccination, wherever it can be done with propriety, Mr. E. prefers going back to Dr. Jenner, as he believes that scarcely one fact or principle of any importance has been added to the plain, unpretending, but luminous account, originally published by him. " In regard to vaccination, the many volumes written on it since his time will be found, on a careful examination, to be little else than mere amplifications or confirmations (always excepting the objurgations of his opponents,) of what was originally said by him."

Mr. Brown observes, that one ground of the doubts originally entertained respecting the efficacy of the vaccine disease was "its possessing no character resembling the disease which it was meant to combat." To this Mr. Edmonston rejoins, very justly, that, "without adverting to several distinguishing marks of similitude between the two diseases, actually traced by Dr. Jenner, it will suffice to assert, what admits of no dispute, that the non-resemblance, even did it exist, militates nothing against its power of combating the antagonist disease." (P. 15.)

The second ground of doubt is its exerting *no* sensible or distinct influence over the human body. The answer to this objection is obvious. In the first place, the vaccine disease *does*, in the majority of cases, exert a sensible influence upon the body; and, secondly, the degree of general derangement produced would form no criterion of its power of preventing small-pox.

We pass over several paragraphs of Mr. Brown's letter, which our author formally, and perhaps unnecessarily, canvasses, as they contain assertions "that might be met by a flat unceremonious negative." Mr. Brown is also more bold than just in his assertion that

"It is now generally granted, that the vaccinated cases are not only more readily influenced by the small-pox contagion, but also in severity, according to the extent of the period from vaccination; and these severe, dangerous, and even fatal cases, have, with very few exceptions, generally occurred at not less than ten years from the period of undergoing the vaccine disease; and there seems an inclination among those only who have been the professed advocates of vaccination to limit the period of increased facility and severity to this distance from vaccination, and that, after that period, there seems no additional tendency to be more strongly influenced by the small-pox contagion." (P. 26.)

Such an opinion may have been entertained by a few hasty and partial opponents of vaccination, but it has never been countenanced by the profession in general. Lord Liverpool is also instructed by Mr. Brown, that

"The reason that the cases of failure do not assume an uniform appearance will be found satisfactorily explained, either from the difference in the severity and mode of application of the small-pox contagion, or from the different extent of influence imparted to the constitution, by the variety in the vaccine phenomena; for, according to the severity and extent of the vaccine phenomena, so is the extent of impression and security imparted to the constitution." (P. 27.)

It is charitable to dismiss this erroneous statement by lamenting the want of knowledge it betrays upon the subject of vaccination. There are few practitioners, we apprehend, who need be guarded against the dangerous practice a concurrence in it would lead to. Nothing could be more injurious to the interests of vaccination than the belief of the necessity of causing a *severe* disease. One perfect and *undisturbed* vesicle is adequate to every purpose of security.

We really cannot follow Mr. E. seriatim through his refutation of Mr. Brown's "ten insurmountable facts." We have glanced at a few of them, and—*ex pede Herculem.*

Having clearly shown that the "facts" are fables,—that they have been built up by very narrow, and not always very liberal views, upon this most important subject,—the author proceeds to the investigation of other opinions and statements scattered through Mr. Brown's letter. The first duty imperatively imposed upon Mr. B. is, that he should specify the sources of the evidence which would substantiate the contents of his letter to Lord Liverpool. He asserts that "the most satisfactory evidence" might be adduced. It is remarkable, however, that it is not brought forward.

Mr. Edmondston makes many judicious observations upon the subject of *Revaccination.* He maintains, and very properly too, that this is a question on which it is absolutely necessary that the public mind should be at once and for ever disabused, whatever be the sentiments entertained of vaccination.

"What purpose can its supporters intend that it should serve? Would they once declare this, the subject might be more effectually grappled with; but I have never yet been able to find out the precise object at which they aim. It will be granted, I imagine, that vaccination must have either a permanent or temporary preventive power. If permanent, the necessity of revaccinating is of course done away with; if temporary, it must be for either a limited or unlimited period. If limited, even with the greatest exactness, every individual must have himself periodically and punctually vaccinated to the end of his days; a result which no system of medical economics could ever render attainable, or even endurable. If indefinite and unfixable, there is an end of the whole question. Dr. Jenner stated that, 'although the cow-pox shields the constitution from the small-pox, and the small-pox proves a protection against its own future poison, yet it appears that the human body is again and again susceptible of the infectious matter of the cow-pox.' This principle, however, I believe he afterwards modified, so far as to consider the phenomena which occur after the first presumed constitutional impression as possessing only a local character." (P. 63.)

It has been a favorite idea with a part of the public, and a few members of the profession also, that it would be well to go back to the cow for occasional supplies of fresh matter in all its original purity. It has been suggested that the virus *may* have undergone a change, from passing through so many constitutions, "it may have degenerated, or become enfeebled, and so forth." Again, the *proofs* of these suppositions are required; for, "under fair circumstances, not the most trivial deviation can be detected between the last vesicle produced and the first case inoculated from the cow by the discoverer himself."

Upon one point we are inclined to differ from Mr. Edmondston. We should certainly wish that, in all cases, one vesicle at least should be allowed to pass through its usual course, without matter being taken from it. We do not think either that the cases are numerous which are vaccinated with only one puncture, and we are pretty confident that the practice of taking matter from a single vesicle is rarely adopted. We presume that the National Vaccine Establishment must have had some grounds for forbidding this practice; and as their opinions must have been formed from more extensive experience than any individual can possess, we should be inclined to conform to their recommendation.

The following passage deserves attention:

"As to the cicatrix, or any other of the phenomena singly, affording a criterion of perfect or imperfect vaccination, much need not be said. It is certain, and must therefore be admitted once for all, that small-pox has succeeded to cow-pox, where the cicatrix has exhibited all the alleged characteristics, while persons have escaped in whom these have been by no means so distinct,—nay, when they have been almost wanting. But what does this amount to? Really nothing. It is equally certain that small-pox has succeeded to variolation where the surface of the body has been pitted all over, while, in cases where not a mark can be traced, it has made no impression; yet we find many respectable authorities reasoning upon the appearance of the cicatrix as on a thing unalterably fixed. Of these I may here cite Dr. George Gregory, who, when speaking of cicatrices, says, 'the proofs of vaccination were distinct and undeniable." If he mean proofs of the act of vaccinating having been performed, he may be right; but I doubt whether he can say that, of perfect constitutional vaccination, he possesses one or more distinct, undeniable proofs? Even of constitutional variolation, I maintain there is no distinct, undeniable proof. What then is the cicatrix worth in either case? Why, it amounts, under any circumstances, to no more than a very strong presumption; and this view of the case is strengthened when we remember that the local phenomena may sometimes be manifested as perfectly when the constitution is not fully influenced as when it is. For any one, then, after examining the cicatrices, to declare that an individual has undergone perfect or imperfect vaccination, is, in my humble judgment, to declare more than there is sufficient warranty for; while the interests of vaccination must suffer in proportion to the degree of confidence assumed." (P. 73.)

The test recommended by Mr. Bryce is considered by the author to be impracticable in its general application, although he admits the ingenuity of the suggestion.

Mr. E. is certainly correct in asserting that cases of small-pox have *not* happened most frequently in those who have been longest vaccinated; neither has the susceptibility to small-pox contagion, nor the tendency to a fatal disease, increasing with the distance from the period of vaccination, been generally observed.

The custom of vaccinating infants within the first month after birth is said to deserve the severest reprobation. "At best, it is justifiable only under the most emergent circumstances, or when small-pox is at the door." A little speculation is hazarded as to the defective organization of the infant at so tender an age. But we would ask, are there any facts tending to show that small-pox has more frequently succeeded to vaccination in those cases where the child has been vaccinated during the first month, than in those where a later period has been chosen?

Mr. E. can perceive no satisfactory grounds (neither can we) for the opinion of Dr. G. Gregory, "that the younger the lymph, the greater the degree of its intensity." The eighth day is usually considered the most proper period for taking lymph, and Mr. E. observes, "that the whole phenomena and pathology of the disease distinctly point to that day, as to the period when the virus is present in its highest state of propagative energy."

Our readers are doubtless aware that Dr. FERGUSON has lately suggested the idea of producing by art the disease termed varioloid, or modified small-pox, by inoculation with both poisons: that is, with cow-pox and small-pox. The object proposed by this plan is to combine the mildness of cow-pox with what is supposed to be the greater security of small-pox. In reference to this practice, Mr. Edmondston observes,

"This theory of Dr. Ferguson either goes upon the principle of keeping alive pure cow-pox and pure small-pox, or it means nothing. Now, it is plain that, supposing the scheme to be carried into effect, the object aimed at cannot be accomplished. For instance, if I inoculate a patient A, I must take the matter from two patients, B and C, the one under genuine cow-pox, the other under genuine small-pox. The effect of this twofold inoculation is not cow-pox, nor is it regular small-pox; but it is, or ought to be, modified small-pox. Here, then, my progress is stopped; for, if I wish to inoculate a second patient D, I dare not do it from A, with his varioloid or modified disease, because that is expressly declared by Dr. F. to be 'dangerous.' What, then, is to become of D, if there be no double supply of matter at hand. I must either have in reserve, or must procure a fresh supply somewhere else. But then, according to the theory, all other practitioners must be similarly situated, and consequently in no better condition to assist me than I am to assist them. Besides, such a thing is necessarily debarred by the doctrine, which enjoins mildness and security, by inducing a varioloid disease. To obtain this, two patients would, in every instance, be required for the inoculation of one by the double method, and thus, by a sort of suicidal operation, the process must speedily destroy itself. We must either go on preserving two stocks of virus, which is impossible, or if possible, is inadmissible by the theory, or the two diseases must be exhausted in the ratio of two to one, and in the last resort we shall have nothing left but the modified or varioloid disease, from which we are told by Dr. F. that it is dangerous to propagate the disease further. Here, then, we should be brought to a stand; for, if we proceed at all, we must of necessity inoculate all the patients that hereafter may be with the virus of this modified or varioloid disease, which, as it is not a hybridous compound, could not be made to preserve its new character, but would be commutable into small-pox in the very first unprotected case, and the whole business would have to be commenced *de novo*." (P. 113.)

If the refutation of Mr. Brown's opinions and statements had been the only object effected by the letter of Mr. Edmondston, we certainly should not have bestowed upon it so much of our attention. His doctrines are so obviously erroneous, his assertions upon the subject of vaccination so directly opposed to the general experience of the profession, that no danger need be apprehended from them. For several years Mr. Brown maintained opinions the very reverse of those he now upholds: he was originally a staunch friend to vaccination. Perhaps he may once more change; and, if he does *not*, the cause of vaccination will never suffer from the want of so fickle a supporter. If he again ventures to break a lance upon the same subject, he may be safely entrusted to Mr. Edmondston, who, in the work we have just noticed, "hits him hard and oft." If he does venture to renew the combat, we shall be astonished at his pertinacity in a bad cause, and lament his want of judgment.

There are many points connected with the subject of vaccination upon which the public, and even many of the profession, entertain the most erroneous views. Upon most of these sources of error, and even mal-practice, the present letter will be found to convey very useful instruction. Mr. Edmondston is a very eloquent and pleasing writer, and, although he sometimes deals very severely with Mr. Brown, we can freely forgive him for the merits of his cause, and for the provocation he has received.

---

*An Essay on the Diseases of the Jaws, and their Treatment; with Observations on the Amputation of a part or the whole of the Inferior Maxilla: tending to prove that such Operations is seldom, if ever, necessary.* With two Plates. By LEONARD KOECKER, Surgeon Dentist, Doctor in Medicine and Surgery; Member of the Medical and Linnean Societies, and of the Academy of Natural Science of Philadelphia; and Author of the "Principles of Dental Surgery," &c. &c. &c.—8vo. pp. 95. T. and G. Underwood, London, 1828.

THE publication of this little work tends to confirm the favorable impression made upon our minds by Mr. KOECKER's previous labours, and places him, in our opinion, in a very prominent situation as a scientific practitioner in the department which he has especially chosen.

In a modest preface, the author, after stating the difficulties with which a foreigner has to contend as a writer, especially when his opinions may be supposed to aim at novelty or originality, continues thus:

"In publishing this small Essay, I may be accused of a presumptuous attempt to treat on a subject which does not belong to my particular province: this, I trust, however, will be deemed erroneous, when it is considered that, although in their later and more complicated stages, the maladies of the jaws require the united aid of general surgery and medicine, they strictly, in their earlier forms, belong to the practice of dentistry, and never would require the assistance of the former, if the latter were judiciously afforded at a proper period.

"There is, moreover, a considerable difficulty in deciding at what period the exclusive treatment of the teeth becomes insufficient, and when the surgical and medical agencies are indispensably required; an inconvenience which can only be removed by affording all branches of the healing art the means of acquiring the most comprehensive views of the history, nature, and causes of the diseases in question." (P. 6.)

Mr. Koecker's Essay commences with some pages of preliminary remarks: these are principally critical, and have for their object to point out the erroneous views formerly entertained, and indeed still inculcated in modern works, upon the causes of many of the maladies of the maxillary bones. We shall not, however, observe the author's arrangement, but commence our extracts at page 28, where we find him making physiological and pathological remarks on the jaws. The diseases of the maxilla, says our author, rarely, if ever, have their origin in the cavity or antrum; but, in every instance where the mucous lining is affected, this will be found to be the consequence of disease of some part of the osseous structure surrounding it: the name, therefore, of disease of the antrum is an incorrect one, and has led to erroneous theory and practice. Both jaws are equally subject to these diseases, but with this difference, that the progress of disease in the upper jaw is facilitated by its greater vascularity and more spongy structure, which at the same time renders a natural palliation or cure of the complaint more common than in the more dense, osseous structure of the inferior maxilla, and also from the situation for the discharge of matter being more inconvenient. From the same circumstances, Mr. K. thinks that cancerous ulcerations more frequently occur in the upper jaw, without much previous tumefaction; whilst in the lower one sarcomatous and osteo-sarcomatous tumors more usually form, terminating fatally by the supervention of carcinoma. These tumors are always to be considered as consequences of other primary affections, and probably the earlier or later occurrence of the tumefaction depends much upon the state of the constitution itself. In the delicate, the progress is slower than in the robust; and this, though protracting the case, retards the period of tumefaction, as, when the disease has arrived at an acute state, it is constantly relieving itself by discharge, and then returns to a chronic form. In strong constitutions, the reverse of this is the case. The former variety of disease is most common in America, the latter in England and in the continent of Europe. These, when properly treated, admit generally of a more successful termination; but if, on the contrary, they be neglected or ill managed, afford the most dangerous varieties of the disease. The following passage we give in the author's own words:

"If art lends its aid to remove the local causes of the disease, nature will soon effect a perfect cure without further assistance. But the constitution being more active in its curative efforts, although incapable of removing the local causes without surgical assistance, the morbid action is liable to be increased by such powerful exertions of nature. If, moreover, these natural efforts are improperly interfered with by the treatment usually applied in cancerous affections, such as the exhibition of henbane, hemlock, mercury, &c. or by operations usually adopted in general surgery, the disease is seldom even palliated, and frequently aggravated by such unnecessary and painful operations, or stimulant remedies, all of which, when applied without a previous removal of the local exciting causes, must naturally augment the disease in the same manner as, though in a greater degree than, the unsuccessful efforts of nature. Hence it is in this state that the disease is more frequently considered incurable, while in fact it is more manageable than in any other: the difference is, that in this the powerful efforts of nature require more judicious attention on the part of the surgeon-dentist than in the other, in which the more passive state of the parts, and greater chemical activity of the matter, give a more distinct indication of the proper curative means.

"In constitutions, however, which are not only suffering from debility, but which are at the same time under the influence of actual disease, or of a general vitiated state of the system, such as is induced or excited by scrofula, scorbutus, syphilis, the abuse of mercury, or powerful narcotic medicines, &c., the diseases of the jaws most frequently proceed rapidly to their greatest extent and fatal termination. They are, moreover, produced often by the slightest causes: sometimes one dead tooth or stump is sufficient to give rise to great inflammation and mortification in the bony structure of the jaw, as well as in the membrane lining the cavity, and to hasten the primary disease through all its different grades; while exposure to great cold or heat, an accidental blow or fall, or any other irritation of a similar kind, acting upon the structures contiguous to parts already symptomatically affected, is quite adequate to excite, at every period of the malady, any of the secondary diseases, such as polypi, or œdematous, sarcomatous, and osteo-sarcomatous tumors and excrescences. Indeed, these secondary tumors may sometimes be observed at a period when the primary affection is so little advanced as entirely to escape surgical observation; and they may proceed to their greatest extent in a period of one or two years, before the idiopathic disease of the bony maxillary structure has had time to proceed to an advanced stage." (P. 33.)

This, which is the most unmanageable form of the disease, requires a combined medical, surgical, and dental management to obtain a favorable result. Thus any treatment which does not involve the removal of the local causes, both primary and secondary, will aggravate, and hurry the disease to its malignant state. The removal of a polypus, or a spongy or bony tumor, will be useless, unless the morbid condition of the jaw be also removed or cured; whilst this must be combined with a due attention to constitutional remedies.

The symptoms of the diseases of the jaw may be soon dismissed: they are similar to those occasioned by dead roots of teeth, or by affections of the alveoli and periosteum, from which they merely differ in degree of violence.

Of the causes of this disease our author treats more at length: they are chiefly those produced by local irritation, aggravated by constitutional derangement, and errors in diet, and even narcotic medicines or mercury, administered previously to the removal of the local disease, will tend to aggravate it; and so indeed will the free exhibition of mercury, from whatever cause. Our author enumerates among the causes of these diseases all operations performed with the intention of destroying the nerve or sensibility of the teeth; and then speaks of the method lately introduced of cutting off the crown of a tooth, in the following words:

"But the operation of breaking or cutting off the crown of painful teeth, which the inventor calls excision, is nothing less than an amputation by violent means, and cannot be adopted from any other cause than a culpable timidity on the part of the patient or the dentist, who are thus led to substitute it for the necessary extraction of the teeth, without even preserving the only useful and essential part, viz. its crown. It unquestionably effects, although not either without pain or so instantaneously as it is asserted, a destruction of the vitality of the remaining roots or stumps, which then become extraneous bodies; the permanent irritation of which, however, must tend to excite disease and induce mortification, not only in adjoining parts, but also in the remaining teeth and gums, not to mention the very great and dangerous irritation produced at the same time upon the whole nervous system.

"Should this be doubted, I beg to refer every medical and surgical reader to a careful examination of the parts, which will evidence the fact; for it will be found that, in a hundred jaws containing roots or stumps, without one single exception, the parts contiguous to the roots exhibit some marks of disease or mortification: unless, indeed, the teeth have been broken after the death of the subjects from which the bones are taken." (P. 42.)

We are next presented with some observations upon Inflammation, Suppuration, and Fistulous Perforations and Abscesses of the Jaw; and these are followed by the detail of an interesting but fatal case of malignant and cancerous affection of the jaw; for the relation of which, however, we have not room: but this we regret the less, because one or two of these cases have already been published.

We must now conclude this article by saying a few words relative to the mode of treating these various affections; but here we cannot do justice to our author, because the relation of the cases and the plan pursued in treating them are so mixed up together that it is impossible to separate them: we shall therefore conclude by inserting, in our author's own words, the general principles of treatment of inflammation and suppuration of the jaws:

"The first and most important indication of treatment in this affection is to remove from the maxillæ, and every part of the whole mouth, whatever irritation may have produced, or which tends to keep up the inflammatory action. This may generally be affected by the extraction—1st, of every dead root and tooth; 2d, of every tooth suffering from complicated caries, or every painful tooth; 3dly, of every large grinder which is deprived of its antagonist; and, 4thly, of every other tooth which is loose, irregular, or situated in any part primarily affected, or in any way capable of acting as a cause of irritation and excitement upon any part of the mouth, or which might be in the least expected to interfere with the exfoliation of the dead parts, or with the complete removal of them.

"All the teeth and roots which constitute the causes of the disease should be removed, if possible, at the same time; for a removal at different intervals will greatly diminish and protract the salutary result of the treatment. This, however, may frequently be deemed impossible, and the dentist must content himself with their removal at different periods. Any treatment, however, without the removal of them altogether, at least in a short space of time, will only be an injurious palliation, and occasion a relapse of the disease, sometimes more violent than the first attack; for the remaining affected teeth will keep up the morbid action, and either lessen or totally prevent the healthy inflammation of the whole mouth, but especially in the parts most extensively affected; whereas a perfect removal of these causes, followed by the greatest cleanliness of the mouth, will frequently effect a cure for the disease, even without any further operation.

"The second indication is to procure the most favorable discharge of the matter, and promote the healthy action of the parts affected by the adaptation of such means as are consistent with correct general surgical principles." (P. 61.)

